# Upregulating Nonneuronal Cholinergic Activity Decreases TNF Release from Lipopolysaccharide-Stimulated RAW264.7 Cells

**DOI:** 10.1155/2014/873728

**Published:** 2014-03-09

**Authors:** Yi Lv, Sen Hu, Jiangyang Lu, Ning Dong, Qian Liu, Minghua Du, Huiping Zhang

**Affiliations:** ^1^Laboratory of Shock and Multiple Organ Dysfunction, Burns Institute, First Hospital Affiliated to the People's Liberation Army General Hospital, 51 Fu Cheng Road, Beijing 100048, China; ^2^Department of Pathology, First Hospital Affiliated to the People's Liberation Army General Hospital, 51 Fu Cheng Road, Beijing 100048, China

## Abstract

Nonneuronal cholinergic system plays a primary role in maintaining homeostasis. It has been proved that endogenous neuronal acetylcholine (ACh) could play an anti-inflammatory role, and exogenous cholinergic agonists could weaken macrophages inflammatory response to lipopolysaccharide (LPS) stimulation through activation of *α*7 subunit-containing nicotinic acetylcholine receptor (*α*7nAChR). We assumed that nonneuronal cholinergic system existing in macrophages could modulate inflammation through autocrine ACh and expressed *α*7nAChR on the cells. Therefore, we explored whether LPS continuous stimulation could upregulate the nonneuronal cholinergic activity in macrophages and whether increasing autocrine ACh could decrease TNF release from the macrophages. The results showed that, in RAW264.7 cells incubated with LPS for 20 hours, the secretion of ACh was significantly decreased at 4 h and then gradually increased, accompanied with the enhancement of *α*7nAChR expression level. The release of TNF was greatly increased from RAW264.7 cells at 4 h and 8 h exposure to LPS; however, it was suppressed at 20 h. Upregulating choline acetyltransferase (ChAT) expression through ChAT gene transfection could enhance ACh secretion and reduce TNF release from the infected RAW264. 7cells. The results indicated that LPS stimulation could modulate the activity of nonneuronal cholinergic system of RAW264.7 cells. Enhancing autocrine ACh production could attenuate TNF release from RAW264.7 cells.

## 1. Introduction

Acetylcholine (ACh), traditionally regarded solely as a neurotransmitter, is synthesized in cholinergic neurons and released via vesicular machinery in response to physiological and pharmacological stimulation. ACh acts through nicotinic and muscarinic receptors in nerves and peripheral tissues. In 1966, Morris reported that ACh was synthesized in the placenta and made the initial description of nonneuronal ACh synthesis [1]. Subsequently, growing evidence indicates that, besides neuronal ACh, a broad variety of nonneuronal cell types throughout the body (such as lymphocytes, macrophages, dendritic cells, adipocytes, keratinocytes, endothelial cells, and epithelial cells) also produce and release ACh and express choline acetyltransferase (ChAT), acetylcholinesterase (AChE), and acetylcholine receptors (AChRs) [2–9]. In 1998, Wessler et al. proposed the concept of   “nonneuronal acetylcholine” [10]. A wide distribution of nonneuronal acetylcholine on multiple tissues or cells suggested that ACh and its receptors played a regulating role in maintaining the homeostasis of these tissues or cells by autocrine/paracrine signaling pathway, or as a participant in the progression of severe pathologies of some diseases [10–13].

Borovikova et al. [14] found that vagus nerve stimulation attenuated the systemic inflammatory response to endotoxin (lipopolysaccharide (LPS)) and developed the concept of “cholinergic anti-inflammatory pathway.” It was observed that vagotomy followed by LPS stimulation resulted in a higher level of tumor necrosis factor (TNF) alpha in serum compared with control animals receiving LPS alone or sham vagotomy [15]. It suggested that, under normal conditions (i.e., in the absence of nicotine), the vagus nerve release of acetylcholine at sites of peripheral tissue innervations provides the source of agonist for the nAChR. The nAChR responsible for this effect was pharmacologically identified to be the *α*7 subunit-containing nicotinic acetylcholine receptor (*α*7nAChR) [16], which was later supported by studies in mice whose *α*7nAChR subunit was genetically eliminated. Wang et al. first reported that acetylcholine or nicotine pretreatment of human peripheral blood mononuclear cells acted through a posttranscriptional mechanism to reduce the amount of TNF present in the media 2 hours following stimulation with the bacterial component lipopolysaccharide (LPS) [16, 17]. Macrophages have complete cholinergic components including ACh, ChAT, AChE, and nAChRs. We want to find out if macrophages autocrine ACh play a regulatory role in maintaining their own homeostasis and inducing hyporesponsiveness to exogenous antigenic stimulation.

Prior exposure of macrophages to minute amounts of LPS causes them to become refractory to subsequent LPS challenge, a phenomenon called “endotoxin tolerance” [18, 19]. The molecular mechanisms underlying endotoxin tolerance remain elusive. Because the inhibition of TNF release in endotoxin tolerant macrophages [20, 21] was similar to that in macrophages treated with cholinergic agonists [14–16, 22], accordingly we hypothesized that macrophages stimulated by minute LPS may modulate the synthesis and release of ACh as well as *α*7nAChR expression via their innate nonneuronal cholinergic system, which were involved in inducing macrophages hyporesponsiveness to continuous or subsequent LPS challenge.

Therefore, in this study we investigated whether LPS challenge affects nonneuronal cholinergic components in RAW264.7 cells (mice peritoneal macrophage line) and whether enhancing autocrine ACh in RAW264.7 cells could ameliorate the TNF release and benumb the response to LPS challenge.

## 2. Materials and Methods

### 2.1. Cell Line

RAW264.7 cells were purchased from the American Type Culture Collection (ATCC TIB-71, Manassas, VA, USA) and maintained in RPMI1640 supplemented with 10% heat-inactivated FBS, 2 mM glutamine and 100 U/mL penicillin, and 100 *μ*g/mL streptomycin (Gibco, Carlsbad, CA, USA). Cell cultures were maintained at 37°C, 5% CO_2_ atmosphere.

### 2.2. Cell Culture and Treatment

RAW264.7 cells were seeded in 12-well tissue culture plates at 10^6^ cells per well and were cultured overnight in RPMI1640 with 10% FBS. Prior to adding LPS or PBS, the cells were washed and replaced with fresh RPMI1640 with 10% FBS. Cells were exposed to lipopolysaccharide (LPS; endotoxin) (*Escherichia coli*, L4130 0111:B4; Sigma, St. Louis, MO, USA) (100 ng/mL) for 20 h or PBS (volume equaled with the added LPS) for 4 h. Cell culture media were collected at the times indicated in the figure legends (PBS, LPS 4 h, LPS 8 h, and LPS 20 h) and centrifuged at 1500 rpm for 5 min to sediment cell debris. The centrifuged media were aliquot and were frozen at −80°C until ELISA analysis was performed.

### 2.3. Determinations of TNF and ACh

TNF and ACh secreted into the media by RAW264.7 cells were determined by enzyme-linked immunosorbent assay (ELISA) (Cusabio Biotech, China) according to the manufacturer's instructions.

### 2.4. Determination of AChE Activity in Culture Medium

AChE activity of culture medium was determined by the method reported by Ellman et al. [23], which is based on the formation of the yellow 5-thio-2-nitrobenzoate anion produced in the reaction between 5,5′-dithiobis-(2-nitrobenzoic acid) (DTNB) and thiocholine, after the AChE-mediated hydrolysis of ACh. The reaction was followed spectrophotometrically by the increase of absorbance at 412 nm. AChE activity of the medium was expressed as *μ*mole per mL. The assay was performed according to the corresponding instruction of the assay kit supplied by Jiancheng Biological Institute (Nanjing, China).

### 2.5. Preparation of Cell Lysates and ChAT Content Analysis

RAW264.7 cells (10^6^/mL) were seeded in 6-well tissue culture dishes (2 × 10^6^ cells per well) and cultured overnight and replaced the medium. Then the cells were incubated with LPS or PBS for different periods of time at 37°C and 5% CO_2_ atmosphere. The cells were rinsed twice with PBS and disassociated with nonenzyme cell detach solution. The cells were collected and centrifuged 5 min at 1500 rpm at 4°C. The cells were washed twice with ice-cold PBS and centrifuged as above. The supernatants were discarded and 1 mL nondenatured cell lysis buffer was added to each sample to lyse the cells. The suspensions were vortexed for 10 s and kept on ice bath for 20 min. The lysates were centrifuged at 12000 rpm for 15 min at 4°C to remove the cell debris, and the supernatants were used to assay ChAT and TNF content with ELISA. The protein concentration of the lysates was determined by bicinchoninic acid (BCA). The reagents used in preparation of cell lysates were purchased from Applygen Technologies Inc. in China.

### 2.6. Flow Cytometric Analysis for *α*7nAChR Expression

Change in the expression of *α*7nAChR on RAW264.7 cells was detected by flow cytometry (FACS). RAW264.7 cells were stimulated with 100 ng/mL LPS for 4 h, 8 h, and 20 h, respectively, and the cells incubated with PBS (volume equaled with the added LPS) for 4 h were used as the control. At the corresponding time points, the cells were rinsed with PBS, disassociated with nonenzyme cell detach solution, and transferred to tubes. After being centrifuged 5 min at 1500 rpm at 4°C and washed with PBS, the cells were fixed in 4% polyformaldehyde/PBS 30 min. Then the cells were washed with PBS for three times and suspended in 0.4 mL PBS. A mouse monoclonal antibody against *α*7nAChR (sc-374284, Santa Cruz Biotechnology Inc., USA) was added in the suspension (1 : 100), incubated overnight at 4°C, followed by washing with PBS, and stained with FITC-conjugated goat anti-mouse IgG (H + L) (EarthOx, USA) for 20 min at room temperature. The cells were washed with 2 mL PBS and resuspended in 0.4 mL PBS. All FACS data were analyzed on 10^6^ cells in FACSCalibur 4-color flow cytometer (BD Bioscience). The surface *α*7nAChR expression level was measured as the percent of positive cells.

### 2.7. RNA Extraction, Reverse Transcription, and Quantitative Real-Time Polymerase Chain Reaction

To determine TNF, ChAT, and AChE mRNA expression level, total RNA was isolated from PBS and LPS-treated RAW264.7 cells using TRIzol reagent (Gibco BRL, USA) according to the manufacturer's instructions. The extracted RNA quality and quantity were determined by measuring absorption at 260/280 nm by spectrophotometer. Reverse transcription was carried out with 2 *μ*g total RNA using the SuperScript II reverse transcription system (Invitrogen, USA) according to the manufacturer's recommendations. Semiquantification of mRNA expression level was performed by real-time PCR using Fast SYBR Green Master Mix (ABI, USA) and a StepOne 7500 Fast (ABI, USA). PCR primers of the different target genes and their products' length are indicated in Table 1. PCR program: 1 cycle of 95°C for 20 seconds, followed by 40 cycles of 95°C for 5 seconds and 60°C for 30 seconds. *β*-Actin was used as a housekeeping gene to normalize the amplification signals of target genes. The relative amounts of PCR product were determined using the standard ΔΔCT method. The dissociation stages, melting curves, and quantitative analyses of the data were performed using ABI instrument software SDS2.1.

### 2.8. RAW264.7 Cells Transfections

RAW264.7 cells were seeded in 12-well tissue culture plates at 5 × 10^4^ cells per well and grown overnight in RPMI1640 supplemented with 10% fetal bovine serum. In the next day, the cells were infected with either empty vector or ChAT expression vector of constructed lentivirus with MOI20 according to the manufacturer's protocol (GeneChem Co., China) for 12 hours. The media were discarded, and the cells were washed twice and replaced with fresh RPMI1640 supplemented with 10% FBS. After being cultured for 72 h, the transfected cells were disassociated and seeded in 24-well tissue plate at 6× 10^5^ cells per well and cultured for 20 h. The media were collected for determining ACh and TNF concentrations with ELISA. Cells lyses, RNA isolation, and RT-PCR were performed as described above. The transfection experiment was performed for three independent times.

### 2.9. ChAT Overexpressed RAW Cells Were Treated with LPS

The transfected RAW264.7 cells were seeded in 24-well tissue plate at 5× 10^5^ cells per well and cultured overnight. The cells were washed and replaced with fresh RPMI1640 with 10% FBS and then treated with LPS (100 ng/mL) or PBS for 4 hours. The media were collected, centrifuged, and aliquoted for determining TNF and ACh secretion. The cells lysates and total RNA extraction were performed as mentioned above. The TNF content in lysates was assayed using ELISA, and TNF mRNA expression level in the cells was quantified by real-time PCR as described in Section 2.6.

### 2.10. Statistical Analysis

The data, expressed as mean ± standard deviation (SD), were analyzed from one of two or three independent experiments. Significant differences were assessed by using one way analysis of variance (ANOVA) followed by least-significant-difference (LSD) test. Differences with *P* < 0.05 were considered statistically significant.

## 3. Results

### 3.1. LPS Stimulation Induces the Enhancement of Both TNF Release and mRNA Expression of RAW264.7 Cells

**Figure 1:**
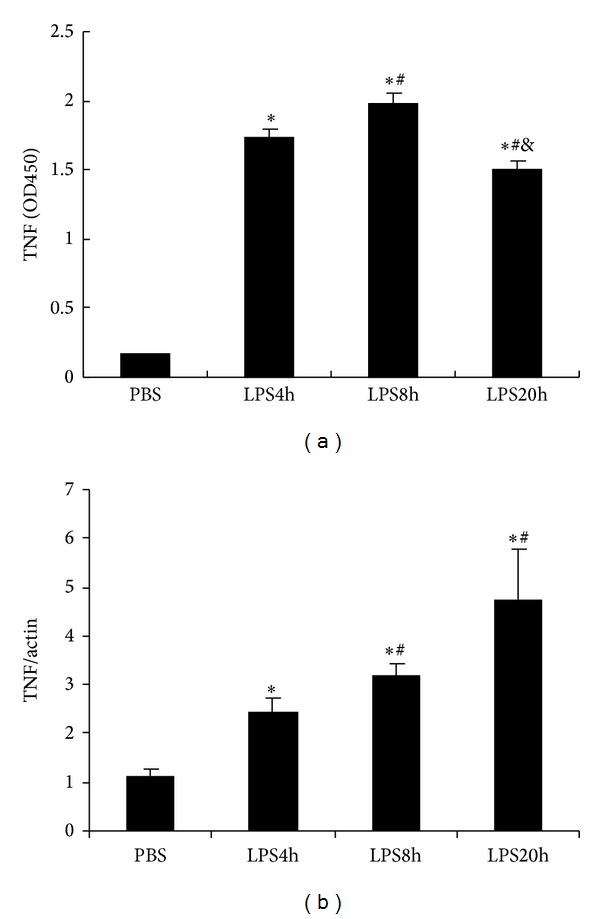
Kinetics of TNF protein and mRNA expression in RAW264.7 cells stimulated with 100 ng/mL LPS. (a) TNF content in the supernatant was determined by ELISA; data are shown as OD450; (b) TNF mRNA expression level was determined by using real-time PCR. *β*-Actin was used as a control gene. The data shown are representative of three independent experiments. All values are expressed as mean ± SD (*n* = 3). *P < 0.05 compared with PBS, ^#^ *P* < 0.05 compared with LPS 4 h, and ^&^ *P* < 0.05 compared with LPS 8 h.

RAW264.7 cells, originating from mice macrophages, could respond to endotoxin and showed overproduction of inflammatory cytokines such as TNF, IL-1, IL-6, and IL-12. When stimulated with LPS (100 ng/mL), RAW264.7 cells released a large number of TNF (Figure 1(a)) and upregulated TNF mRNA expression level (Figure 1(b)). However, the increase of TNF release did not synchronize with that of its mRNA expression level: the amount of TNF release arrived at the peak following LPS stimulation for 8 hours, and subsequently it was decreased; TNF mRNA expression level was gradually enhanced with time-dependent manner during 20 h LPS incubation.

### 3.2. LPS Stimulation Modulated ACh Production and Upregulated *α*7nAChR Expression Level of RAW264.7 Cells

**Figure 2:**
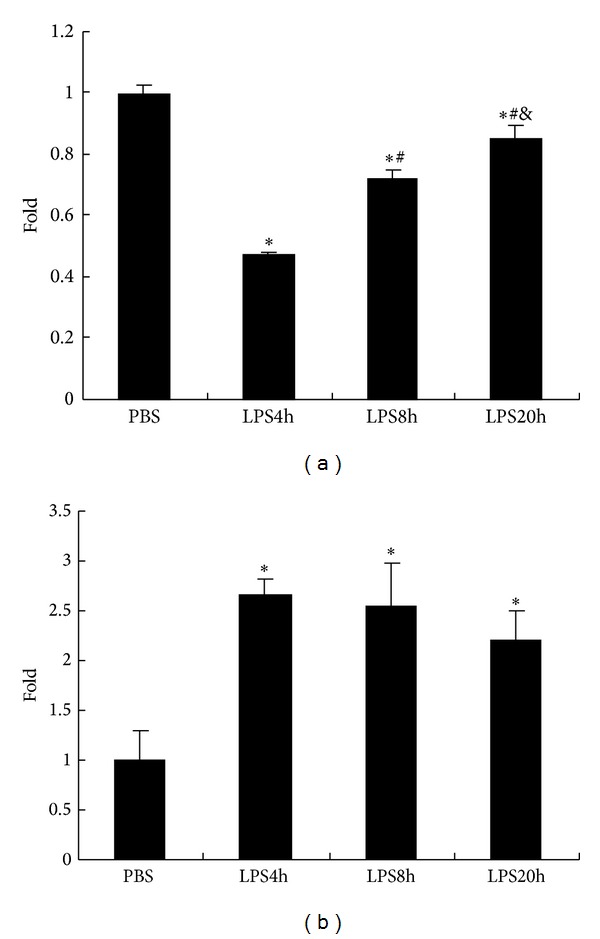
LPS stimulation reduced ACh release and upregulated *α*7nAChR expression of RAW264.7 cells. (a) ACh secretion in the supernatant of RAW264.7 cells incubated with LPS (100 ng/mL). ACh secretion decreased from RAW264.7 cells challenged with LPS for 4 h and then gradually recovered along with the LPS incubated time. (b) The rate of *α*7nAChR positive cells was increased in LPS stimulated RAW264.7 cells, which was maintained at a high level during 20 h LPS exposure. The data are expressed as fold increases compared to unstimulated cells (PBS group). All values are expressed as mean ± SD (*n* = 3), *P < 0.05, compared with PBS, ^#^ *P* < 0.05, compared with LPS 4 h, and ^&^ *P* < 0.05, compared with LPS 8 h. The data shown are representative of two individual experiments.

It has been known that prior exposure of macrophages to minute amount of endotoxin for 18–20 h could make them become refractory to subsequent endotoxin challenge, a phenomenon called “endotoxin tolerance.” We supposed that the innate nonneuronal cholinergic members in macrophages may be involved in inducing their tolerance to LPS overstimulation. So, we analyzed the change in ACh production and its ligand *α*7nAChR expression of RAW cells stimulated by LPS for 20 h. We observed that, in RAW264.7 cells, ACh secretion was significantly dropped following 4 h LPS exposure and then gradually increased by time-dependent fashion (Figure 2(a)). *α*7nAChR expression level of RAW264.7 cells was greatly enhanced following LPS stimulation and had been kept at high level during a period of 20 h LPS incubation (Figure 2(b)). It was shown that the increase of both ACh secretion and *α*7nAChR expression went with decrease of TNF release when RAW264.7 cells were incubated with LPS for 20 h but without relation to TNF mRNA expression fashion.

### 3.3. Effect of LPS Stimulation on ChAT Content in Lysates, AChE Activity in Culture Supernatants, and Their mRNA Expression Levels in RAW264.7 Cells

**Figure 3:**
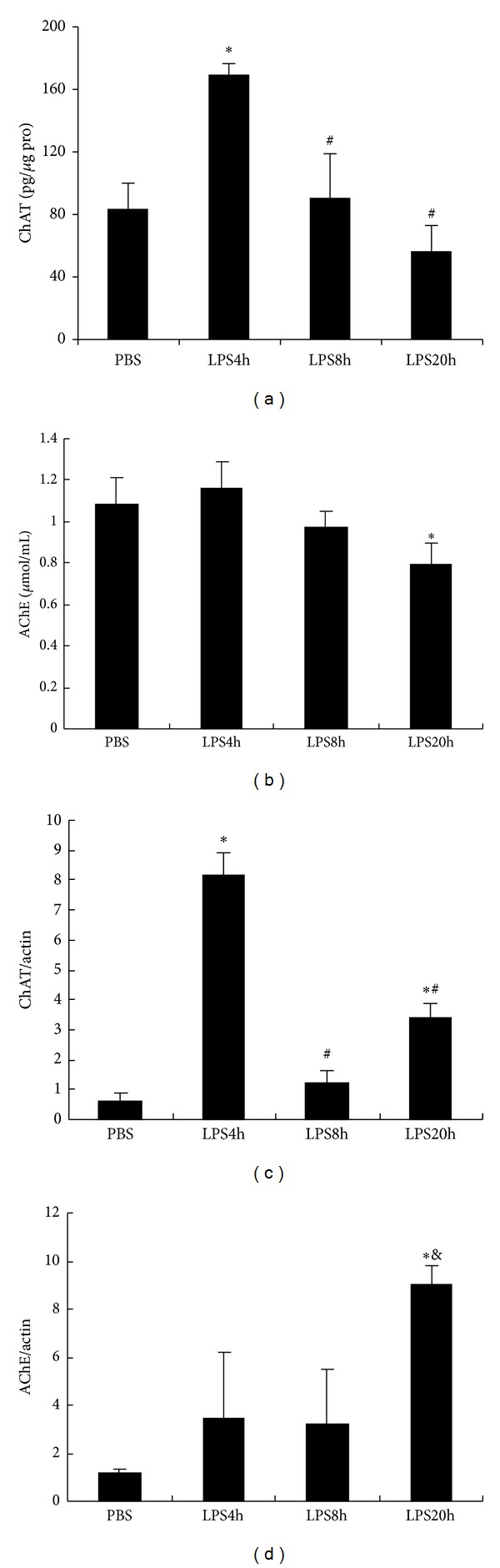
ChAT content within RAW264.7 cells, AChE concentration in the supernatant, and their mRNA expression level at the indicated time points of LPS exposure. (a) ChAT protein content in RAW264.7 cells lysates was detected by ELISA and the protein concentration of the lysates was determined by BAC method. The data were presented as pg ChAT per *μ*g protein. (b) AChE activity in supernatants of LPS treated RAW264.7 cells at the indicated time points, which was determined by the method of Ellman. (c) ChAT mRNA and (d) AChE mRNA expression levels in LPS treated RAW264.7 cells, which were determined by real-time PCR. Values are expressed as the ratio of ChAT or AChE to *β*-actin. The data are representative of three individual experiments and presented as mean ± SD (*n* = 3), *P < 0.05, ^#^ *P* < 0.05, and  ^&^ *P* < 0.05 when compared with PBS, LPS 4 h, and LPS 8 h groups, respectively.

ACh, ChAT (an enzyme catalyzing ACh synthesis), AChE (an enzyme catalyzing ACh degradation), and AChRs constitute an integrated nonneuronal cholinergic system in RAW264.7 cells. Based on altered ACh production and upregulated *α*7nAChR expression in LPS treated RAW cells, we further analyzed changes in ChAT contents and AChE activity of LPS stimulating RAW cells. Generally speaking, the increase of ACh concentration is accompanied with the augmentation of ChAT content or the weakness of AChE activity. Curiously, we observed an unexpected phenomenon: ChAT content in RAW cells was greatly enhanced at LPS challenge 4 h, followed by a gradual decrease, which showed a negative change with autocrine ACh production. ChAT mRNA expression level was upregulated in RAW264.7 cells during 20h LPS stimulation. AChE activity in culture medium and mRNA expression level of RAW264.7 cells were not altered significantly during 8 h LPS incubation. But at 20 h following LPS stimulation, AChE activity in the supernatant was lowered obviously and its mRNA expression level was elevated significantly (Figure 3).

### 3.4. Upregulated Expression of ChAT by Transfecting Lentiviral Carried ChAT Gene Could Promote Autocrine ACh Synthesis and Suppress TNF Release of RAW264.7 Cells

**Figure 4:**
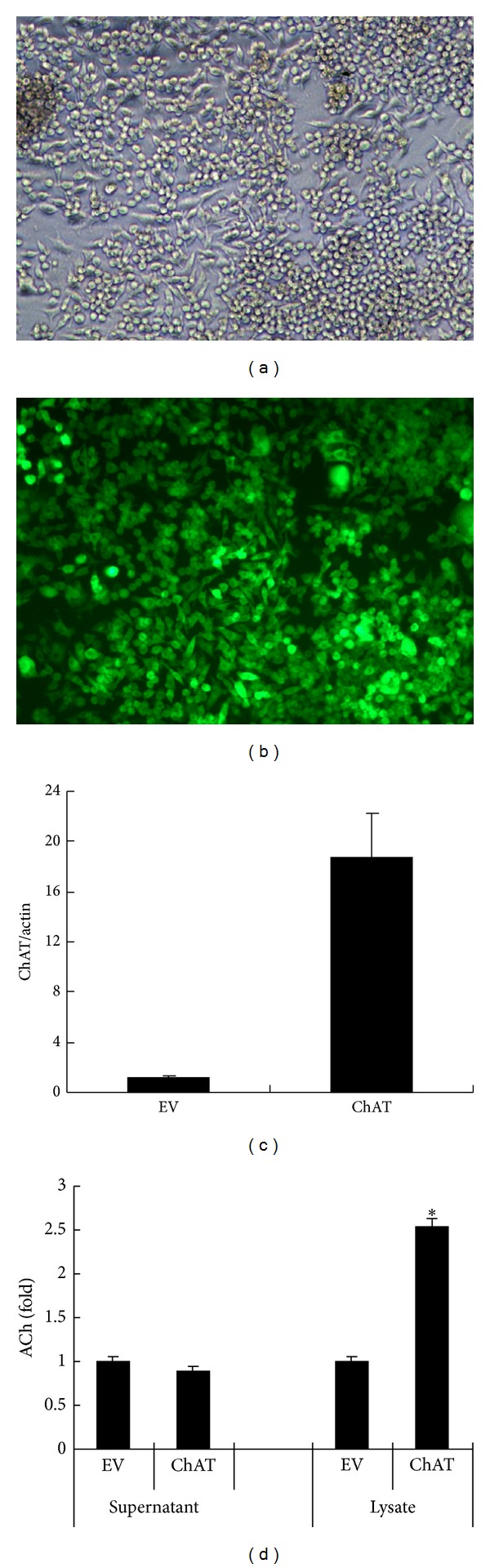
ChAT mRNA expression level and ACh secretion level in RAW264.7 cells transfected with empty vector (EV) or ChAT expression vector (ChAT). (a) RAW264.7 cells transfected by ChAT expression vector under inverted phase contrast microscopy (×100), which showed a good state. (b) GFP expression was more than 80% RAW264.7 cells infected by ChAT expression vector, under fluorescence microscopy (×100). (c) ChAT mRNA expression level was upregulated largely in RAW264.7 cells transfected by ChAT expression vector. (d) The amount of ACh in the supernatant and lysate of ChAT overexpressed RAW264.7 cells; the determination of ACh was performed by ELISA. Data are representative of three independent experiments and expressed as mean ± SD (*n* = 3), *P < 0.05 when compared with EV group.

To explore whether autocrine ACh could ameliorate TNF release from RAW264.7 cells, we employed the method of transfecting lentiviral carried ChAT gene into RAW264.7 cells to make the infected cells overexpressing ChAT and overproducing ACh. The data in Figure 4 showed that the cells transfected with ChAT expression vector (ChAT) could overexpress ChAT mRNA and produce more ACh than the control cells transfected with empty vector (EV) (Figures 4(c) and 4(d)). ACh production in the transfected cells (in the lysate) was greatly elevated; however, there was no significant difference in the amount of ACh secretion in culture supernatants between overexpressed ChAT cells and the control cells.

### 3.5. Increasing Autocrine ACh of RAW264.7 Cells Suppresses TNF Release but Upregulates TNF mRNA Expression

**Figure 5:**
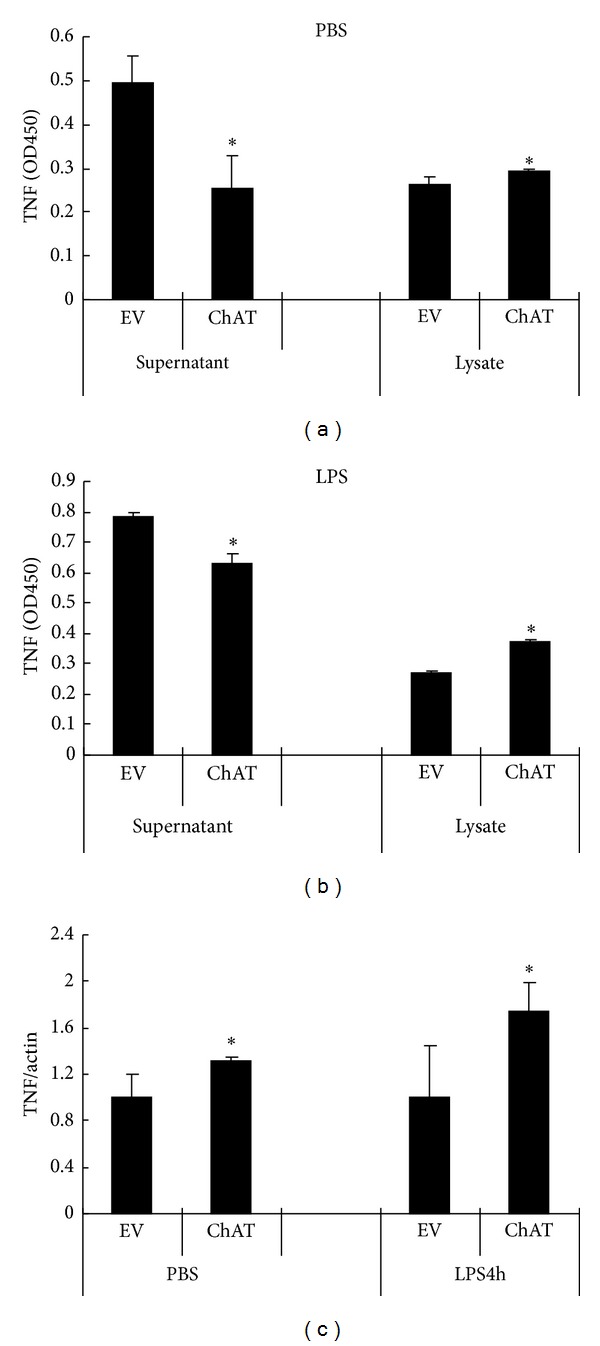
TNF protein and mRNA expression levels in empty vector (EV) and ChAT expression vector (ChAT) infected RAW264.7 cells. TNF content in supernatant and in lysates of empty vector and ChAT expression vector infected RAW264.7 cells following incubation for 4 h: (a) untreated cells (PBS instead of LPS). (b) LPS treated cells. Compared with empty vector infected cells, TNF content in the supernatant decreased and in the lysate was increased in ChAT overexpressed RAW264.7cells. The amount of TNF both in supernatant and in lysate was detected by ELISA and shown as OD450. (c) TNF mRNA expression level in ChAT gene transfected RAW264.7 cells was higher than that in empty vector transfected RAW264.7 cells, both in LPS treated and untreated cells. TNF mRNA expression level was determined by real-time PCR. *β*-Actin was used as the control gene. The data shown are representative of three independent experiments and presented as mean ± SD (*n* = 3). *P < 0.05 when compared with EV group.

We next observed the effect of increasing autocrine ACh production on TNF release of RAW264.7 cells.  Figure 5 shows TNF content in the culture supernatant and lysate, as well as TNF mRNA expression level of both ChAT vector and empty vector transfected RAW264.7 cells. In the presence or absence of LPS, the quantity of TNF in the supernatant was less in ChAT transfection RAW264.7 cells than in the control cells. However, it is puzzling why TNF content in lysate and TNF mRNA expression abundance were enhanced when ACh production was increased in ChAT overexpression RAW264.7 cells (Figure 5(c)). The results suggested that the increase of autocrine ACh production may inhibit TNF release rather than reduce TNF mRNA expression and protein synthesis.

## 4. Discussion

The present study suggests that continuous LPS stimulation could modulate the nonneuronal cholinergic autocrine activity of RAW cells. It is evidenced by changes of ACh secretion and upregulated expression of cholinergic ligand *α*7nAChR in LPS stimulated murine macrophages (RAW264.7 cells). Moreover, increasing autocrine ACh production via upregulating ChAT gene expression in RAW264.7 cells could suppress TNF release in presence or absence of LPS. The results indicated that macrophages could establish the adaptation to LPS stimulation partly via their innate nonneuronal cholinergic autocrine loop.

Animals pretreated with a low dose of endotoxin have a markedly reduced mortality when rechallenged with a normally “lethal” dose of endotoxin [24, 25], a phenomenon termed “endotoxin tolerance.” In humans, endotoxin tolerance was developed during five consecutive LPS administrations as demonstrated by the attenuated release of proinflammatory cytokines on the fifth day and was associated with less leukocyte and endothelial activation [26]. This refractoriness to LPS is considered to be an adaptation to prevent overstimulation from the continuous exposure to LPS [27, 28]. Nevertheless, it is now evident that the early proinflammatory phase of the sepsis is immediately followed by an anti-inflammatory response that rapidly results in an immunosuppressive state. Immunosuppression is believed to be the critical factor for the patients' secondary infection and to increase the risk of mortality [29–31]. The development of immunosuppression is associated with negative feedback regulation and the so-called endotoxin or microbial tolerance. Various mechanisms have been proposed for endotoxin tolerance. Among them are the downregulation of the LPS-receptor TLR4 [32], loss of tyrosine phosphorylation of TLR4 [33], and decreased recruitment of the adaptor protein MyD88 to TLR4 or suppressed interaction between MyD88 and the kinase IRAK-1 [34, 35]. Furthermore, a shift from transcriptionally competent NF-kB heterodimers (p50/p65) to inactive homodimers (p50/p50) was associated with the tolerance state [36]. The upregulation of inhibitory proteins (such as IRAK-M, SOCS-1, and SHIP1) [37–39] and anti-inflammatory cytokines (IL-10 and TGF) [40] induced by low dose microbial stimulation also is involved in the generation of the hyporesponsive state. Recently, microRNAs in LPS-induced gene reprogramming urge a reevaluation of endotoxin tolerance [41]. Tracey's group proposed that exacerbated release of TNF and other proinflammatory cytokines could be suppressed by the efferent vagus nerve based cholinergic anti-inflammatory pathway [14, 17] and further demonstrated that *α*7nAChR is an important component underlying the anti-inflammatory efficacy [16]. The discovery of the cholinergic anti-inflammatory pathway provides not only a new strategy for targeting excessive inflammatory diseases but also a novel clue for exploring the mechanisms inducing macrophages tolerance to LPS stimulation.

In this study, we observed that there are ACh, *α*7nAChR, ChAT, and AChE in RAW264.7 cells, which constitute a complete nonneuronal cholinergic system. ACh secreted in the supernatant dropped sharply when incubation of RAW264.7 cells with LPS for 4 h subsequently increased in a time-dependent fashion during the period of 4 h–20 h LPS exposure. In contrast, *α*7nAChR expression on RAW264.7 cells, one of the cholinergic members, was upregulated significantly and maintained at a high level during 4 h–20 h LPS incubation. The relative increased autocrine ACh and upregulated expression of *α*7nAChR could strengthen the cholinergic activity in LPS challenged RAW264.7 cells through promoting the interaction between Ach and *α*7nAChR. It has been identified that prior exposure of macrophages to minute LPS for 12 h–29 h could lead to hyporesponsiveness to subsequent LPS challenge [20, 42]; meanwhile endogenous ACh and exogenous cholinergic agonists could decrease inflammatory cytokines release from LPS stimulated macrophages [14, 15, 22]. We infer that sustained LPS stimulation could upregulate the nonneuronal cholinergic activity in the challenged RAW264.7 cells, which was likely to play a role in limiting the excessive inflammation of primed RAW264.7 cells. This kind of negative feedback regulation would contribute to the restoration of homeostasis of the primed cells. Nevertheless, the continuing negative feedback effect* in vivo* is likely to be one of the mechanisms mediating immunosuppression in sepsis.

Physiologically, the balance between synthesis and degradation of ACh depends on the coordination of ChAT and AChE expression. We assume that change of ACh secretion induced by LPS should associate with upregulation of ChAT expression or downregulation of AChE activity. However, we accidently found that ChAT content was changed negatively with autocrine ACh production during 20 h LPS incubation and AChE activity was not changed obviously during 8 h LPS stimulation. Interestingly, at 20 h after LPS stimulation, there was cooperativity among ACh secretion, ChAT content, and AChE activity, which was presented as increased ACh release and reduced AChE activity in the supernatant and augmented ChAT content in the lysate of RAW264.7 cells. The correlation between AChE and ChAT levels was observed by Kaufer et al. [43]. They found that the ACh increase due to forced swimming stress or inhibitors of the acetylcholine-hydrolysing enzyme AChE could result in markedly upregulated expression of AChE mRNA, as well as downregulated expression of ChAT mRNA in cortex or sagittal corticohippocampal slices of mice. Their results indicate that acute cholinergic stimulation promotes selective bidirectional changes in the expression of genes regulating acetylcholine metabolism. In the present study, the coordinated regulation of AChE and ChAT in sustained exposure to LPS could support the balance between ACh synthesis and degradation in RAW264.7 cells and therefore make the cells adapt to LPS challenge. The regulation of AChE expression was complex and multiple. Waiskopf et al. reported that fluoxetine as an antidepressant could intercept the LPS-induced decreases in intracellular AChE [44]. LPS exposure induced overexpression of the AChE-targeted microRNA-132 [45, 46] and AChE antisense ODN mEN101 [47] also have been found to suppress AChE expression. The induced expression of AChE possesses dual effects. On the one hand, the stressful induced AChE directly hydrolyzes Ach to promote inflammation. On the other hand, the intracellular AChE could interact with the nuclear factor kappa B-activating intracellular receptor for activated C kinase 1 to take part in anti-inflammation [44]. In addition, the induced AChE could selectively contribute to cellular apoptosis through its noncatalytic properties [48, 49]. The therapeutic agents can thus be targeted to the AChE protein, its encoding mRNA transcripts, or the regulator, opening new venues for therapeutic interference with immune system diseases.

TNF is one of potent proinflammatory cytokines induced by LPS challenge [50], as well as most probably the best marker of endotoxin tolerance as assessed by its dramatically reduced production following an LPS challenge in tolerant animals and macrophages [51, 52]. We examined the kinetics of TNF production and mRNA expression level in LPS treated RAW264.7 cells. It was found that, in the duration of 20 h exposure to LPS, TNF mRNA expression level was gradually increased in a time-dependent manner. However, the amount of TNF release was raised greatly at 4 h and arrived at the maximum at 8 h, followed by decrease at 20 h following LPS stimulation. The increase of TNF mRNA expression did not synchronize with that of TNF release. Since cholinergic anti-inflammatory pathway inhibits TNF expression at the posttranscriptional level [14, 52], we infer that the weakened TNF release at 20 h of LPS stimulation was associated with the increases of autocrine ACh release and *α*7nAChR expression. Nahid et al. have also reported that, in supernatant from cultured LPS-stimulated THP-1 cells, TNF started to appear within 2 h and reached a maximal level at 4 h of stimulation followed by gradually decreased starting at 8 h. They found that TNF increased up to 4 h and then decreased gradually implicating a negative correlation with miR-146a progression [41]. Hamano et al. found that nicotine suppressed the expression of CD14, toll-like receptor 4, intercellular adhesion molecule 1, B7.1, and CD40 on monocytes and the production of TNF via *α*7nAChR [53]. These data indicated that multiple regulating pathways, including nonneuronal cholinergic system, may take part in ameliorating the RAW264.7 cells' response to LPS overstimulation. It is likely that autocrine ACh mediates macrophages' hyporesponsiveness to continuous LPS stimulation through downregulating LPS signaling molecules.

In order to explore the effect of autocrine ACh on TNF release from RAW264.7 cells, ChAT expression vector of lentivirus was constructed and transfected into RAW264.7 cells. The infected RAW264.7 cells could upregulate ChAT expression, which resulted in increasing ACh production and decreasing TNF release from both LPS-stimulated and unstimulated RAW264.7 cells. Unexpectedly, both protein content and mRNA level of TNF were higher in ChAT expression vector infected cells than in empty vector infected cells. This means that the increase of ACh is able to suppress TNF release but unable to inhibit TNF mRNA expression and its protein synthesis. These data agree with Borovikova et al. finding that the anti-inflammation of cholinergic agonists is operated at the posttranscriptional level [14].

In summary, we described here for the first time that continuous LPS stimulation could upregulate autocrine ACh production and *α*7nAChR expression of RAW264.7 cells, which may play a primary role in inducing the hyporesponsiveness of macrophages to LPS stimulation. Increasing autocrine ACh could effectively ameliorate TNF release from RAW264.7 cells. These findings establish the autoregulatory effect of nonneuronal cholinergic system on RAW 264.7 cells.

## Figures and Tables

**Table 1:** Sequences of primers used for real-time quantitative PCR analysis.

Gene symbol	Primer sequences	PCR product length (bp)
TNF*α*	F: ATGGGAAGGGAATGAATCCACC R: GTCCACATCCTGTAGGGCGTCT	281

ChAT	F: GTCTCTGAATACTGGCTGAATG R: TGGTGTCTTGGAAGTGCTG	106

AChE	F: ACTACCGAGTGGGAACCTTTGGC R: CCTGTGGAAGAGGCTCCTGCTG	224

*β*-Actin	F: GCGTGACATCAAAGAGAAGC R: AGCACTGTGTTGGCATAGAG	270

F: forward, R: reverse.
